# A 10-year trend in piglet pre-weaning mortality in breeding herds associated with sow herd size and number of piglets born alive

**DOI:** 10.1186/s40813-020-00182-y

**Published:** 2021-01-04

**Authors:** Yuzo Koketsu, Ryosuke Iida, Carlos Piñeiro

**Affiliations:** 1 School of Agriculture, Meiji University, Higashi-mita 1-1-1, Tama-ku, Kawasaki, Kanagawa 214-8571 Japan; 2 PigCHAMP Pro Europa S.L., c/Santa Catalina 10, 40003 Segovia, Spain

**Keywords:** Piglets, Herd size, Pre-weaning mortality, Year trend

## Abstract

**Background:**

Piglet pre-weaning mortality (PWM) is one of the biggest problems regarding sow performance and piglet welfare. Recently, PWM has increased in some countries, but it is not known if there are similar increases in other countries, nor whether increased PWM is related to either increased numbers of piglets born alive (PBA) or to sow herd size. So, the objectives of the present study were 1) to explore the trend in PWM in Spanish sow herds over a recent 10-year period, along with related measurements such as PBA, stillborn piglets, herd productivity and herd size; and 2) to examine the relationships between PWM and the related measurements.

**Methods:**

Herd-level annual data from 2007 to 2016 for 91 herds in Spain were abstracted from a sow database compiled by a veterinary consultancy firm that asked client producers to mail data files on a regular basis. The database software automatically calculated herd-level PWM (%) as follows: the total number of piglets born alive to a sow completely weaned during a year (TPBA) minus the total number of piglets weaned by the completely weaned sow during the year divided by TPBA × 100. All the statistical analyses were performed by using SAS University Edition. A growth curve model was applied to incorporate correlations for all of the observations arising from the same farm.

**Results:**

Over the 10 years, herd means of PWM (standard deviation) increased from 11.9 (4.1) % to 14.4 (3.2) %, and mean PBA increased by 1.9 pigs. Mean age of piglet death during lactation increased by 3.8 days, and later years were significantly associated with herd size and the number of piglets weaned per sow per year (PSY; *P* <  0.05). Higher PWM was associated with more PBA, more stillborn piglets and small-to-mid herds (lower than the median size: < 570 sows; *P* <  0.05). Also, there was a significant interaction between the herd size groups and PBA for PWM (*P* <  0.05): as PBA increased from 9 to 14 pigs, PWM increased by 9.6% in small-to-mid herds, compared with an increase of only 6.6% in large herds (> 570 sows). Furthermore, as PWM decreased from 18 to 8%, herd productivity measured as PSY increased by 2.2 pigs in large herds, compared with only 0.6 pigs in small-to-mid herds.

**Conclusion:**

Large herds were better than small-to-mid herds at alleviating the association between increased PBA and increased PWM. Also, the relationship between decreased PWM and increased herd productivity was improved more in large herds than in small-to-mid herds.

## Introduction

Piglet pre-weaning mortality (PWM) is one of the key components in the productivity tree of piglets weaned per sow per year (PSY) when PSY is used as an integrated measurement of reproductive performance in sow herds [1–3]. Over the last decade the herd-level mean PWM in the U.S.A. has increased [4]. There are various possible reasons for increasing PWM. For example, it is known that increased PWM is associated with more piglets born alive (PBA) [1, 5, 6], and that genetic improvements in the swine industry over the last few decades have significantly raised PBA [2, 7]. Also, herd-level data analysis has shown that stillborn piglets, later weaning and smaller herd size are also possible factors for high PWM [8–10]. For example, more stillborn piglets have been associated with higher PWM and lower weaning weight [4, 10, 11]. In contrast, late weaning has only been associated with piglets living a few more days in the farrowing room before dying [8, 12], and so it is not clear if late weaning would be directly related to increased PWM. With regard to herd size, a study found that between 2010 and 2014 small herds in Spain had higher PWM than large herds [13]. Also, another study reported that that large herd size was associated with decreased sow longevity [14]. Therefore, it is possible that herd-level factors could affect PWM because large herds have more advanced facilities, more human resources and a higher level of genetic improvement than small herds [12, 15].

Furthermore, it has been widely reported that most piglet deaths occur at 0–1 days after birth [1, 9, 16]. However, it is possible that recent improvements in management practices to cope with increasing PBA and an increased number of low birth weight piglets [16, 17] might alter the age at which piglets die. However, no studies have looked at recent trends in the age of piglet death, nor quantified the relationships between PWM and related herd performance measurements such as PBA and PSY in sow herds. Furthermore, although a study suggested recent increases in PWM in the U.S.A., there have not been any recent studies on PWM trends in another of the major pig producing country, namely Spain. Therefore, the objectives of the present study were to 1) explore the recent trends in PWM, age of piglet death, PBA and other performance measures, 2) examine the association between PBA and PWM, and 3) assess the association between PWM and herd productivity measured as PSY using 10-year herd-level data from Spanish sow herds.

## Materials and methods

### Studied herds and their herd-level data

A veterinary consultancy firm (PigCHAMP Pro Europa S.L. Segovia, Spain) has requested all client producers to mail their data files on a regular basis and has accumulated a sow database. In the summer of 2017, 91 Spanish herds that had 10-year records from 2007 to 2016 were chosen from the database, and their retrospective herd-level annual data for these years were abstracted from the data files.

Between 2007 and 2016, most of the studied herds used farrowing crates in farrowing barns. Also, the lactation and gestation diets in the herds were formulated using cereals (barley, wheat and corn) and soybean meal. Replacement gilts in the herds were either purchased from breeding companies or were home-produced through internal multiplication programs.

### Definitions and categories

Annual PWM values (%) from 2007 to 2016 were calculated by the recording software (PigCHAMP, Ames, U.S.A.) using the following equation: the total number of piglets born alive to a sow completely weaned during a year (TPBA) minus the total number of piglets weaned by the completely weaned sow during the year divided by TPBA × 100. Also, the herds were categorized into two groups (large and small-to-mid herds) based on the 50th percentile of the mean herd size over the 10 years (570 sows). The 50th percentile was chosen as the cut-off point to objectively categorize the studied herds into two groups.

Additionally, herd-years was defined as the number of herds times the number of years containing each herd’s annual data. The studied herds were initially chosen because they were thought to have full 10-year records, so the expected herd-years was 910 (91 herds × 10 years). However, some herds had missing or unreliable data in some years, so these herd-years were excluded (see next section).

### Recording by producers of PWM and age of piglet death

There is a place in the software where producers have the option to record the date that a piglet died and the number of piglet deaths. However, sometimes producers did not record this data or did not record it correctly. As a result, 97 of the 910 herd-year records were excluded: 49 (5.4%) herd-year records were excluded because they had no recorded age for piglet death, and another 48 herd-year records (5.3%) were excluded because the recorded mean age of piglet death was the same as the mean weaning age, suggesting inaccurate recording of the data.

There are two types of PWM data: a herd-level value calculated by the software, and an individual value for each sow derived from the piglet deaths for each sow recorded in the software by the producer. The herd-level PWM calculated by the software was used for all PWM analyses. However, for the analysis on age of piglet deaths, we used the death events recorded by the producers because there was no herd-level software calculation for the age of piglet death.

The two PWM values were compared for herd-level internal consistency. The mean PWM calculated by the software was 12.8 (3.6 standard deviation: SD) % ranging between 1.9 and 24.8%, whereas the mean PWM recorded by the producers was 11.2 (3.9 SD) % ranging between 1.2 and 22.4%. In the dataset, the proportion of individual sow PWM values recorded by the producers was 87.5% of the PWM values calculated by the software. Pearson correlation coefficients between the two types of PWM for each of the 10 years ranged between 0.60 and 0.77 (*P* <  0.01).

### Statistical analysis

All analyses were conducted with SAS University Edition (SAS Inst. Inc., Cary, NC, U.S.A.). Pearson correlation analysis between PWM and other measurements was performed for each of the 10 years using the CORR procedure. Also, a Chi-square test was performed to examine whether or not the frequency distributions of age of piglet death differed between years. The associations were considered significant when the *P*-value was < 0.05.

A growth curve model with random intercept and slope was applied using the MIXED procedure to incorporate correlations for all of the observations arising from the same farm [18, 19], using an unstructured covariance matrix for the random effects and years as continuous variables. So, Model 1 was constructed using the linear year and squared year expression to examine whether there was an increasing or decreasing 10-year trend for PWM and the other key measurements. A cubed year expression was examined only when the squared year expression was significant. We assumed the relationships between the year and examined variables were non-linear.

When sow herd size and age of piglet death were examined as outcome variables, the data were first converted using a square root transformation because those data were not normally distributed. Model 2 used repeated measures analysis to compare PWM and other performance parameters between the two herd size groups. The model included herd size groups and year as fixed effects.

Model 3 examined the associations between PWM or age of piglet death and possible explanatory factors. The model included the following fixed effects, PBA, squared PBA, herd size groups, year, squared year, the number of stillborn piglets and six two-way interactions between the four main factors (PBA, herd size groups, year and the number of stillborn piglets). Two possible factors (i.e. weaning age and gestation length) were not included in Model 2 because the correlations between these two factors and PWM were not consistent over the 10 years (Additional file 1). Also, in preliminary analysis, those two factors were not found to be significant in Model 3 (*P* > 0.10).

Model 4 examined the associations between PWM and either the number of piglets weaned or PSY. It included PWM, squared PWM, herd size groups, year, squared year and three two-way interactions between PBA, herd size groups and year as fixed effects. When PBA and PWM were used as explanatory variables they were centered at the grand means (12.032 pigs and 12.78%, respectively). The adequacy of the model assumptions for the random effects was checked by visual inspection of normal-probability plots [20].

## Results

**Fig. 1:**
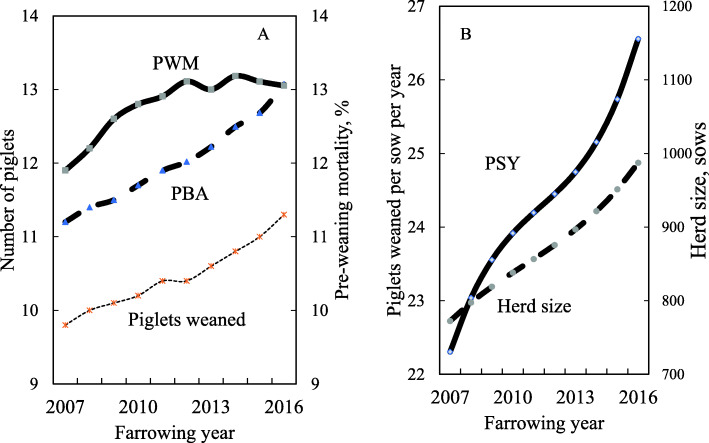
10-year change in 91 herds for (**a**) mean pre-weaning mortality (PWM), the number of piglets born alive (PBA) and the number of piglets weaned, and (**b**) sow herd size and piglets weaned per sow per year (PSY)

Mean PWM increased from 11.9 (4.1 SD) % in 2007 to 13.1 (3.2 SD) % in 2016, and PBA increased from 11.2 (0.8 SD) to 13.1 (1.1 SD) piglets over the same period (Table 1; Fig. 1a). Mean sow herd size (median) also increased over the 10 years from 742 (463) to 957 (650) sows, and PSY increased from 22.2 to 26.4 pigs (Fig. 1b).

**Table 1:** Means (standard deviation) in four selected years (2007–2016), and estimates (Es) ^a^ of year effects (standard error: SE) on pre-weaning mortality and related performance measurements

	Year				Year effect (Y)	
					Y	Y x Y
Measurement	2007	2010	2013	2016	Es (SE)	Es (SE)
Piglets born alive	11.2 (0.83)	11.7 (0.87)	12.2 (0.85)	13.1 (1.06)	0.0758 (0.0236)	0.0107 (0.0019)
Stillborn piglets	1.03 (0.56)	1.04 (0.50)	1.02 (0.49)	1.04 (0.51)	−0.0174* (0.0100)	0.0021 (0.0008)
Piglets weaned	9.8 (0.68)	10.2 (0.68)	10.6 (0.68)	11.3 (0.91)	0.0433 (0.0204)	0.1954 (0.0068)
Pre-weaning mortality, %	11.9 (4.08)	12.8 (3.49)	13.0 (3.66)	13.1 (3.19)	0.3304 (0.1103)	−0.0194 (0.0091)
Age at piglet death, days^b^	2.5 (3.97)	3.2 (4.14)	4.8 (4.24)	5.6 (5.12)	0.1506 (0.0493)	0.0012* (0.0038)
Piglets weaned per sow per year	22.2 (2.88)	23.9 (2.22)	24.5 (2.36)	26.4 (2.96)	0.1882 (0.1029)	0.1954 (0.0068)
Sow herd size	742 (723)	816 (761)	873 (764)	957 (830)	0.2349 (0.1111)	0.0155 (0.0079)

^a^Es of year effects (SE) were obtained from statistical models. The models included the year effect only as fixed effects

^b^The statistical models were applied to square root-transformed values

All estimates shown in the Table were found significant (*P* <  0.05) unless indicated as * (*P* > 0.05)

Table 1 also shows the linear and quadratic expressions of year were found significant for PBA, the number of stillborn piglets, the number of piglets weaned, PSY and sow herd size (*P* <  0.05). Additionally, the linear expression of year was found significant for age of piglet death (*P* <  0.05). However, the cubed year expression was not significant for any measurement (*P > 0.10*).

**Fig. 2:**
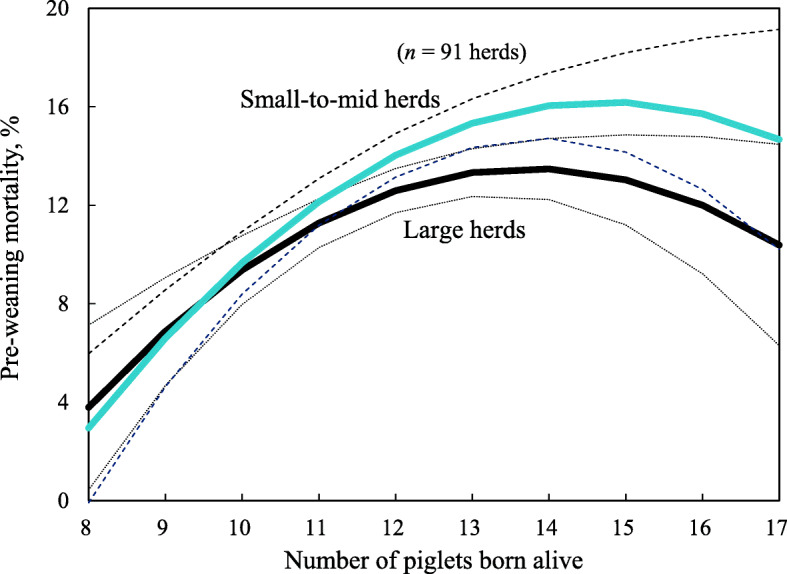
Predicted pre-weaning mortality for different numbers of piglets born alive. Dotted lines show 95% confidence intervals

More PBA, more stillborn piglets and small-mid herds were associated with higher PWM (*P* <  0.05; Table 2). Also, there was a significant two-way interaction between PBA and herd size group for PWM (*P* <  0.05). Figure 2 shows the association between PBA and PWM, using Model 3 containing PBA and year as fixed effects. As PBA increased from 9 to 14 pigs, PWM in small-to-mid herds increased by 9.5% whereas it only rose by 6.6% in large herds (Fig. 2).

**Table 2:** Estimates (standard error: SE) and *P*-values of piglets born alive and sow herd size in the mixed-effects model for pre-weaning mortality in 91 herds

Effects	Pre-weaning mortality (%)	
	Estimate (SE)	*P*-value
Intercept (SE)^a^	12.388 (0.625)	< 0.01
Piglets born alive	1.217 (0.366)	< 0.01
Piglets born alive squared	−0.202 (0.079)	0.01
Sow herd size group	1.845 (0.779)	0.03
Sow herd size group x Piglets born alive	0.577 (0.290)	0.04
Year	0.090 (0.147)	0.96
Year x Year	−0.020 (0.012)	0.05
Year x Piglets born alive	−0.012 (0.052)	0.77
Year x Sow herd size group	−0.014 (0.102)	0.04
Stillborn piglets	2.621 (0.686)	< 0.01
Piglets born alive x Stillborn piglets	0.059 (0.275)	0.82
Year x Stillborn piglets	0.112 (0.101)	0.30
Sow herd size group x Stillborn piglets	−0.471 (0.633)	0.46

^a^SEs were obtained from the statistical model

There were some differences between the two herd size groups, with large herds having lower PWM, more piglets weaned, higher farrowing rates and more PSY than the small-to-mid herds (*P* <  0.05; Additional file 2). However, there was no significant difference between the two herd size groups for PBA (*P* = 0.45).

**Fig. 3:**
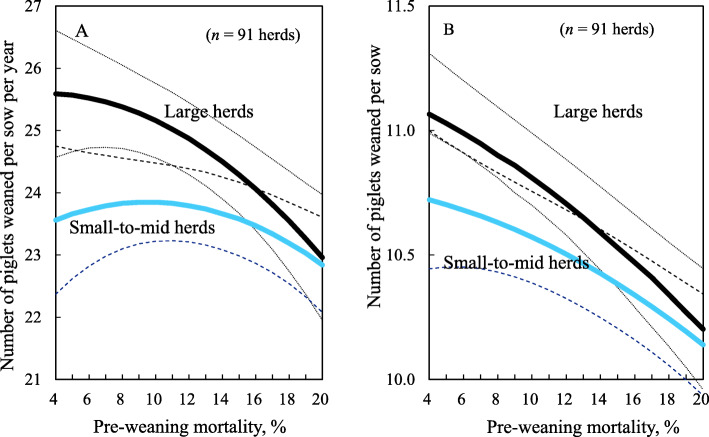
Predicted numbers of (**a**) piglets weaned per sow per year and (**b**) piglets weaned per sow, at different pre-weaning mortality rates. Dotted lines show 95% confidence intervals

Lower PWM was associated with more piglets weaned and more PSY (*P* <  0.05; Table 3). Also, there was a significant two-way interaction between PWM and herd size group for PSY (*P* = 0.04) but not for piglets weaned (*P* = 0.14). Figure 3 shows the associations between PWM and either PSY or the number of piglets weaned. With regard to PSY, as PWM decreased from 18 to 8%, PSY increased by 1.8 pigs in large herds but only by 0.6 pigs in small-to-mid herds (Fig. 3a). In contrast, the increase in the number of piglets weaned as PWM decreased from 18 to 8% was similar for both herd size groups, increasing by 0.4–0.6 pigs (Fig. 3b).

**Table 3:** Estimates (standard error: SE) and P-values of pre-weaning mortality (PWM) and sow herd size in the mixed-effects model for the number of piglets weaned and piglets weaned per sow per year (PSY) in 91 herds

Effects	Piglets weaned		PSY	
	Estimate (SE)	*P*-value	Estimate (SE)	*P*-value
Intercept (SE)^a^	10.1090 (0.1215)	< 0.01	22.5403 (0.4211)	< 0.01
PWM	− 0.0610 (0.0128)	< 0.01	− 0.1483 (0.0605)	< 0.01
PWM x PWM	−0.0011 (0.0009)	0.21	0.0093 (0.0046)	0.04
Sow herd size group	−0.1988 (0.1630)	0.23	−0.2279 (0.5288)	0.66
Sow herd size group x PWM	0.0175 (0.0120)	0.14	0.1197 (0.0582)	0.04
Year	0.0590 (0.0233)	0.01	0.3164 (0.1033)	< 0.01
Year x Year	0.0076 (0.0015)	< 0.01	0.0143 (0.0080)	0.06
Year x PWM	0.0010 (0.0017)	0.59	−0.0056 (0.0082)	0.49
Year x Sow herd size group	0.0017 (0.0225)	0.94	−0.1365 (0.0732)	0.06

^a^SEs were obtained from the statistical model

**Fig. 4:**
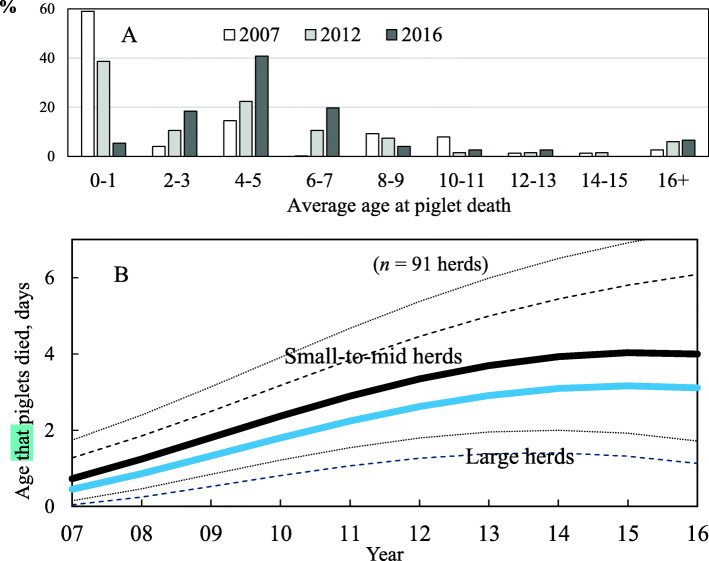
**a** Frequency distributions for age at piglet death in 2007, 2012 and 2016, and **b** predicted age at piglet death in different years. Dotted lines show 95% confidence intervals

Mean age of piglet death increased from 2.5 (4.0 SD) to 5.6 (5.1 SD) days over the 10 years (Table 1), with a significant difference between years in the frequency distribution of piglet death age (*P* <  0.01; Fig. 4a). There were significant linear and quadratic year expressions for age of piglet death in the mixed-effects model (*P* <  0.05; Table 4). Also, there was an interaction between the year and herd size group for age of piglet death (*P* = 0.05). Over the 10 years, the age of piglet death in small-to-mid herds increased by 3.3 days, whereas in large herds it increased by only 2.7 days (Fig. 4b).

**Table 4:** Estimates (standard error: SE) and *P*-values of piglets born alive and sow herd size group in the mixed-effects model for age of piglet death in 91 herds

Effects	Ages of piglets died (%)	
	Estimate (SE)	*P*-value
Intercept (SE)^a^	0.4731 (0.4417)	0.26
Piglets born alive	− 0.3351 (0.2121)	0.10
Piglets born alive squared	−0.0955 (0.0402)	0.02
Sow herd size group	−0.0762 (0.4710)	0.87
Sow herd size group x Piglets born alive	−0.0347 (0.1430)	0.80
Year	0.3585 (0.0879)	< 0.01
Year x Year	−0.0170 (0.0053)	< 0.01
Year x Piglets born alive	0.1220 (0.0245)	< 0.01
Year x Sow herd size group	0.0061 (0.0570)	0.90
Stillborn piglets	−0.8707 (0.3640)	0.92
Piglets born alive x Stillborn piglets	−0.8851 (0.7457)	0.16
Year x Stillborn piglets	−0.0491 (0.0551)	0.37
Sow herd size group x Stillborn piglets	0.2414 (0.3146)	0.44

^a^SEs were obtained from the statistical model

## Discussion

Our study clearly demonstrates that mean PWM, PBA, piglets weaned and PSY increased between 2007 and 2016 in the studied herds. Another study covering a similar time period has reported a significant increase in PBA associated with genetic improvement [2]. Therefore, it appears that genetic progress in the swine industry has led to recent significant increases in PBA and related performance [2, 7].

Our study also showed that more PBA was associated with higher PWM, which is consistent with previous reports in Thailand, Canada and Japan [6, 8, 9]. Additionally, our study clearly showed that the relationship between PBA and PWM differed depending on herd size. For example, as PBA increased from 9 to 14 pigs, PWM in small-to-mid herds increased by 9.5%, compared with an increase of only 6.6% in large herds. So, the increased PWM in large herds was approximately 40% lower than that in small-to-mid herds over the same PBA range. Also, large herds had lower PWM and more piglets weaned than small-to-mid herds. These results suggest that large herds can alleviate the association between increased PBA and increased PWM better than small-to-mid herds. The reasons for this could be that large herds have more advanced facilities than small-to-mid herds, such as well-designed farrowing crates and milk replacer feeders [12, 21]. Also, large herds are likely to have more human resources and a high level of genetic improvement than small-to-mid herds [15]. In addition, our results indicate that PWM is not likely to increase to 20% or higher even if PBA increases to 15 or more pigs, regardless of herd group.

Our study also showed that the average age of piglet death increased by approximately 4 days over the 10 years, with many herds delaying piglet death from the first 0–1 days to 4–5 days after the start of lactation. While current practices such as assisting colostrum intake and split nursing can help small and low birth weight piglets survive and grow [22–25], in some cases these practices may simply delay the inevitable, and such piglets born with low body weights or intrauterine growth retardation [17] may tend to die later in mid-lactation.

The association between more stillborn piglets and higher PWM is consistent with previous studies that reported more stillborn piglets being associated with decreased 21-day litter weights [11], more occurrences of uterine prolapse [26], more abortions at subsequent pregnancy [27] and higher culling risks in sows [28]. These negative associations may suggest that sows with more stillborn piglets tend to be fed in herds with poor hygiene or live in herds with health problems such as porcine reproductive or respiratory syndrome virus [29].

Our study also showed that a 10% decrease in PWM was associated with an increase in PSY of 1.8 pigs in large herds but only with an increase of 0.6 pigs in small-to-mid herds, even though there was little difference between the large and small-to-mid herds in the number of piglets weaned (0.6 vs. 0.4 pigs, respectively). This difference in the associations between PWM and PSY and between PWM and piglets weaned can probably be explained by the fact that sows in large herds have better fertility performance, such as higher farrowing rates and more litters farrowed per sow per year.. Therefore, in addition to decreasing PWM, it is critical for small-to-mid herds to improve the fertilty performance of their sows and increase their herd productivity, including measures such as PSY.

With regard to the limitations of this study, the relationships found between PWM and other variables should be interpreted as associations, not as evidence of causality because our study is a retrospective observational study and not a controlled experiment. Also, the results may not be applicable to all Spanish herds because the studied herds were not randomly selected across from the whole population.

Additionally, our study used the herd as the observation unit to explore the relationship between PWM and other reproductive performance measures over 10 years. We believe that herd-level analysis is appropriate because of herd-level changes in management, health conditions and genetics that might have occurred over such a long period. However, it was not possible to perform multivariate analyses to account for any variation due to parity, season, other possible factors or the herd-entry year of sows, and any interactions with these factors. Also, our results might show a herd-level association between variables which might be different from the association that exists at the sow-level [30]. However, even with these limitations, our longitudinal study provides veterinarians and producers with useful information about the recent trend in PWM, and the relationships between PWM, PBA, herd size and herd productivity.

## Supplementary Information

**Additional file 1.** Correlation coefficients of relationships between pre-weaning mortality and performance-related measurements.

**Additional file 2.** A comparison between two herd groups for pre-weaning mortality and other reproductive performance measurements in 91 herds.

## Data Availability

The dataset analyzed during the current study is not publicly available because producers’ privacy could be compromised.

## Abbreviations

PBA: piglets born alive
PSY: the number of piglets weaned per sow per year
PWM: pre-weaning mortality
