# Supplementary material for: A 10-year trend in piglet pre-weaning mortality in breeding herds associated with sow herd size and number of piglets born alive
Source: Porcine Health Manag. 2021 Jan 4;7:4. doi: 10.1186/s40813-020-00182-y (PMC7784010; doi:10.1186/s40813-020-00182-y)
Supplement: Supplementary file 1 — Additional file 1. Correlation coefficients of relationships between pre-weaning mortality and performance-related measurements. [file 40813_2020_182_MOESM1_ESM.docx]

Additional file 1. Correlation coefficients of relationships between pre-weaning mortality and performance-related measurements

| Measurement | 2007 | 2008 | 2009 | 2010 | 2011 | 2012 | 2013 | 2014 | 2015 | 2016 |
| --- | --- | --- | --- | --- | --- | --- | --- | --- | --- | --- |
| Farrowing performance |  |  |  |  |  |  |  |  |  |  |
| Piglets born alive | 0.40** | 0.41** | 0.41** | 0.44** | 0.34** | 0.36** | 0.43** | 0.18 | 0.27* | 0.30** |
| Stillborn piglets | 0.27** | 0.40** | 0.17 | 0.30** | 0.38** | 0.33** | 0.20 | 0.39** | 0.40** | 0.45** |
| Gestation length, days | -0.05 | -0.02 | -0.11 | -0.05 | -0.08 | 0.04 | 0.01 | 0.20 | 0.24* | 0.21* |
| Weaning performance |  |  |  |  |  |  |  |  |  |  |
| Weaning age, days | 0.12 | 0.16 | 0.08 | 0.10 | 0.19 | 0.24* | 0.21* | 0.17 | 0.13 | 0.16 |
| Population measurements |  |  |  |  |  |  |  |  |  |  |
| Sow inventory | -0.23* | -0.24* | -0.15 | -0.21 | -0.22* | -0.24* | -0.23* | -0.35** | -0.24* | -0.17 |
| Culling rate | 0.12 | -0.15 | -0.05 | 0.22 | -0.02 | -0.02 | -0.02 | -0.12 | -0.23* | -0.11 |
| Mortality | 0.12 | 0.22* | 0.24* | 0.07 | 0.09 | 0.09 | 0.09 | -0.01 | -0.11 | 0.03 |

*indicates 0.01<P<0.05; ** indicates P<0.01.
