# Supplementary material for: A 10-year trend in piglet pre-weaning mortality in breeding herds associated with sow herd size and number of piglets born alive
Source: Porcine Health Manag. 2021 Jan 4;7:4. doi: 10.1186/s40813-020-00182-y (PMC7784010; doi:10.1186/s40813-020-00182-y)
Supplement: Supplementary file 2 — Additional file 2. A comparison between two herd groups for pre-weaning mortality and other reproductive performance measurements in 91 herds. [file 40813_2020_182_MOESM2_ESM.docx]

Additional file 2. A comparison between two herd groups for pre-weaning mortality and other reproductive performance measurements in 91 herds

|  | Large herds | Small-to-mid herds |
| --- | --- | --- |
| Measurement | Mean (standard error: SE) ^*^ | Mean (SE) ^*^ |
| Number of herds | 45 | 46 |
| Sow herd size | 1370 (86) ^a^ | 340 (86) ^b^ |
| Pre-weaning mortality (PWM), % | 11.9 (0.5)^b^ | 13.5 (0.5)^a^ |
| Piglets born alive | 12.1 (0.12) | 11.9 (0.12) |
| Weaning age, day | 23.2 (0.4) ^b^ | 25.0 (0.4) ^a^ |
| Piglets weaned | 10.8 (0.1) ^a^ | 10.5 (0.1) ^b^ |
| Litters per sow per year | 2.35 (0.02) ^a^ | 2.28 (0.02) ^b^ |
| Piglets weaned per sow per year | 24.9 (0.3) ^a^ | 23.5 (0.3) ^b^ |
| Age at piglet death | 6.0 (0.8) | 6.9 (0.8) |
| Farrowing rate, % | 84.3 (0.6) ^a^ | 81.5 (0.6) ^b^ |
| Weaning-to-first-mating interval, day | 6.44 (0.15) | 6.82 (0.15) |

*Mean (SE) values and P-values were estimated by using statistical models.

^ab^ Different superscripts within a row represent significant differences in means (*P* <  0.05).
